# Evaluation of LHP^® ^(1% hydrogen peroxide) cream versus petrolatum and untreated controls in open wounds in healthy horses: a randomized, blinded control study

**DOI:** 10.1186/1751-0147-53-45

**Published:** 2011-06-30

**Authors:** Tamás Tóth, Hans Broström, Viveca Båverud, Ulf Emanuelson, Elisabeth Bagge, Tommy Karlsson, Kerstin Bergvall

**Affiliations:** 1 University Animal Hospital Equine Clinic, Swedish University of Agricultural Sciences, SLU, Box 7040, SE-750 07 Uppsala, Sweden; 2 Dept of Clinical Sciences, Swedish University of Agricultural Sciences, SLU, SE-750 07 Uppsala, Sweden; 3 Dept of Bacteriology, National Veterinary Institute, SVA, SE-751 89 Uppsala, Sweden

## Abstract

**Background:**

Treatment and protection of wounds in horses can be challenging; protecting bandages may be difficult to apply on the proximal extremities and the body. Unprotected wounds carry an increased risk of bacterial contamination and subsequent infection which can lead to delayed wound healing. Topical treatment with antimicrobials is one possibility to prevent bacterial colonization or infection, but the frequent use of antimicrobials ultimately leads to development of bacterial resistance which is an increasing concern in both human and veterinary medicine.

**Methods:**

Standardized wounds were created in 10 Standardbred mares. Three wounds were made in each horse. Two wounds were randomly treated with LHP^® ^or petrolatum and the third wound served as untreated control. All wounds were assessed daily until complete epithelization. Protocol data were recorded on day 2, 6, 11, 16, 21 and 28. Data included clinical scores for inflammation and healing, photoplanimetry for calculating wound areas and swab cytology to assess bacterial colonization and inflammation. Bacterial cultures were obtained on day 2, 6 and 16.

**Results:**

Mean time to complete healing for LHP^® ^treated wounds was 32 days (95%CI = 26.9-37.7). Mean time to complete healing for petrolatum and untreated control wounds were 41.6 days (95%CI = 36.2-47.0) and 44.0 days (95%CI = 38.6-49.4) respectively. Wound healing occurred significantly faster in LHP^® ^wounds compared to both petrolatum (p = 0.0004) and untreated controls (p < 0.0001). There was no significant difference in time for healing between petrolatum and untreated controls. Total scores for bacteria and neutrophils were significantly (p < 0.0001) lower for LHP^® ^treated wounds compared to petrolatum from day 16 and onwards. *Staphylococcus aureus *and *Streptococcus zooepidemicus *were only found in cultures from petrolatum treated wounds and untreated controls.

**Conclusions:**

Treatment with LHP^® ^reduced bacterial colonization and was associated with earlier complete wound healing. LHP^® ^cream appears to be safe and effective for topical wound treatment or wound protection.

## Background

Treatment and protection of wounds in horses can be challenging. Protective bandages can be difficult to apply especially on the proximal extremities and body. Unprotected wounds carry an increased risk of bacterial infection which can result in delayed wound healing [1]. Topical antimicrobial treatment may prevent bacterial colonisation and infection but frequent use of antibiotics can induce allergic side effects [2] and it is a root cause of the development of bacterial resistance, which is an increasing concern in both human and veterinary medicine [3-5]. Methicillin-resistant *Staphylococcus (S.) aureus *has long been a recognised problem in human medicine and is an emerging problem in veterinary equine practice as well, involving both horses and veterinary personnel [6-10]. Restricted use of antibiotics in the prevention of wound infections is thus of great importance.

Various antiseptics have been advocated in wound management. Hydrogen peroxide is one of them that has long been used in both human and veterinary medicine [11,12]. In water solution it rapidly decomposes into water and oxygen in contact with organic tissue and has a short acting time. In a cream formula hydrogen peroxide is included in a stabilized form that allows a slow degradation and a prolonged effect [13-15].

A cream containing 1% hydrogen peroxide has been reported to be effective and safe for topical treatment in human impetigo contagiosa, where the main pathogen is *Staphylococcus aureus *and less commonly β-haemolytic streptococci [15]. In that double-blinded, fucidic acid controlled study, the hydrogen peroxide cream showed excellent antibacterial effect and eliminated most of the bacteria from the skin. The authors suggested that the cream could be useful against other superficial skin infections as well. Hydrogen peroxide cream has also been successfully used in treatment of human acne vulgaris [16,17]. None of these studies reported any adverse effects such as skin irritation or allergic reaction in human patients. Hydrogen peroxide cream (1,5% - 3%) has also been used in an animal model where it increased circulation in ischemic ulcers and surrounding skin in guinea pigs [18].

White petrolatum has also been found to be a valuable alternative for topical wound treatment in humans. It has been used in several studies and was demonstrated to be beneficial in wound healing [2,19-21]. White petrolatum was less tissue-irritating and protected both open and primarily closed wounds from infection as efficiently as ointments containing gentamicin or bacitracin [2,19].

We hypothesized that LHP^®^cream and white petrolatum could be beneficial for topical wound treatment and/or wound protection. To our knowledge the effect of LHP^®^cream and petrolatum on equine wounds has not yet been reported. The aims of the study were to investigate effect of LHP^® ^cream and petrolatum on wound healing time and bacterial colonisation when applied to open skin wounds in the horse.

## Methods

The study involved ten adult, healthy, non-pregnant standardbred mares. The mean age of the horses was 14.8 years (median 15.0 years, range 9-20 years). Their mean weight was 520.3 kg (median 510 kg, range 466 - 562 kg). The horses were kept in the same stable under identical housing conditions (bedded on straw, fed hay and oats and water ad libitum) and turned out on the same paddock for the same period during daytime. The horses had been dewormed according to current routines. No medication except sedation was allowed two weeks prior to inclusion. All horses received a general clinical examination by the same examiner upon inclusion. The study was performed during January and February to avoid the fly season. The study protocol was approved by the Research Animal Ethics Committee of the Swedish Board of Agriculture (C290/9) prior to commencement.

Detomidine hydrochloride (0.02-0.04 mg/kg, IV) combined with butorphanol tartrate (0.01-0.02 mg/kg, IV) was used for sedation and analgesia. Two 6 × 6 cm areas were shaved at standardized locations on each side of the neck (one on the left and one on the right side) and a third area of the same size was prepared identically, approximately 8 cm cranial to the previously described location. Five horses had the cranial wound randomly chosen on the left side and five horses had it on the right side. The wound locations were assigned by lot-drawing by the surgeon. The areas were aseptically prepared using clorhexidine digluconate soap and chlorhexidine digluconate alcohol rinse according to standard clinical protocol. A subcutaneous injection of 2 ml mepivacaine hydrochloride without adrenalin was administered for local anesthesia at the centre of each area. Full thickness skin wounds were created in the centre of each shaved area, with a 2 cm diameter circular punch manufactured for this study (Ångström Laboratory, Uppsala University, Sweden). All wounds were created by the same surgeon. The horses were identified by using numbers (1-10) and the wounds by letters (A-C).

All wounds were unprotected and left to heal by second-intention. Each wound was uniformly cleansed with three swabs soaked with sterile 0.9% sodium chloride two times daily. The paired wounds on the corresponding sides (left and right) of the neck were randomly assigned to treatment with LHP^®^cream (LHP^®^, Bioglan Pharma AB, Malmö, Sweden**) or petrolatum (protective white vaseline [ACO HUD AB, Stockholm, Sweden]) by lot-drawing. The wounds were treated topically with three ml LHP^®^cream or three ml petrolatum twice daily after wound cleansing until they were considered healed. The third wound was left without treatment and served as untreated control. The wounds were handled identically and disposable gloves were changed between each lesion. The treatments were carried out by the same technicians and remained blinded to the investigators. All horses were examined once daily for general status, fever, unusual behavior and also local signs of wound infection. In case of severe swelling, discharge and fever indicating wound infection treatment with antimicrobials would be initiated and the horse excluded from further study after recording the treatment. Horses indicating discomfort and pain (biting, sore to the touch) would receive treatment with nonsteroidal anti-inflammatory drugs and would also be excluded from further study after recording of the treatment.

All wounds were evaluated for protocol data on day 2, 6, 11, 16, 21 and 28. A scoring system (scores 0 - 4) for swelling, sensitivity to the touch, discharge, granulation and epithelization was used for subjective evaluation. In this system lower summarized scores were associated with more advanced healing and a complete wound healing would receive score 0. After subjective evaluation bacterial cultures on day 2, 6, and 16, and sterile swabs for cytology on day 2, 6, 11, 16, 21 and 28 were taken from all wounds. The samples for bacterial culture and cytology were taken from the unprepared wound areas approximately 12 hours following last treatment and before wound cleansing. Swabs were rolled on the wound surface in each wound. Bacterial swabs were rolled perpendicularly crossing the wound surface 4 times and roll-lines did not overlap. Swabs for cytology were taken following bacteriological samples and sampling followed similar pattern except that the roll-lines were horizontal.

Samples for bacterial culture were taken with a sterile ESwab (Copan Innovation Ltd, Brescia, Italy) moistened with three drops of sterile 0.9% sodium chloride. The swabs were transported to the laboratory in liquid Amies transport media (Copan Innovation Ltd) and were cultured within six hours on 5% bovine blood agar (blood agar base no. 2 [Oxoid.CM0271, Basingstoke, Hampshire, England] 40 g/L; citrated bovine blood [Håtunalab, Bro, Sweden] 50 ml/L) plates and bromcresol purple lactose agar (balanced peptone, 10 g/L [LabM]; NaCl, 5 g/L [Merck]; sodium ammoniumphosphate, 1 g/L [Merck]; Lab Lemco Powder, 4 g/L [Oxoid]; Agar no. 2, 10 g/L [LabM]; lactose solution 20%, 50 mL/L [SVA]; bromcresol purple solution 1.6%, 1 mL/L [SVA]) plates. Bacterial growth was examined after incubation for 24 and 48 h at 37°C. Colonies were identified by colony morphology, Gram stain and biochemical fermentation according to standard laboratory procedure. The cultures were all blinded and analyzed at the same laboratory. Bacterial growth on the plates were classified as no growth, sparse (*n *< 20), moderate (20 to 100), or profuse (> 100) on the basis of the number of colonies on the agar plates. Growth of *β*-haemolytic streptococci, *Staphylococcus aureus*, *Pseudomonas aeruginosa *and *Klebsiella pneumoniae *was always considered to be of significance. Other bacterial isolates were typed and considered as significant if growth was in pure culture or dominating on the agar plate. From the same sample, two bacterial species might be isolated and typed. When colonies of different bacterial species were detected on one agar plate and not considered as significant (see above), growth was described as nonspecific mixed flora.

Cytology slides were examined by a blinded investigator. The slides were stained with Hemacolor (Merck, Darmstadt, Germany) and examined at 1000 × (high power field, HPF). The mean number of bacteria (cocci and rods) were calculated per 10 HPF and assigned a score from 0-4 (0 = absent, 1 = <5, 2 = 5-10, 3 = 11-25, 4 = >25). Neutrophils were counted and mean number per 10 HPF were scored from 0-4 (0 = absent, 1 = <1, 2 = 1-5, 3 = 6-10, 4 = >10).

**Figure 1:**
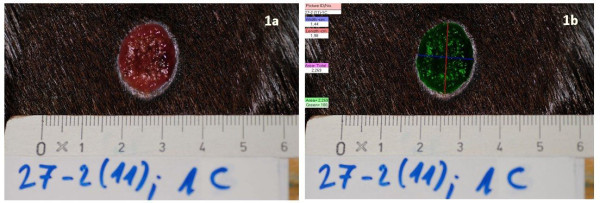
**Wound area measurement by using digital photoplanimetry**. A wound photographed on day 11 (1a) and the wound area (also length and width) is measured by using digital photoplanimetry software (1b).

After sampling, the wounds were cleansed by using sterile swabs soaked in 0.9% sodium chloride as previously described and all wounds were photographed. All photographs were taken with the same digital camera (Nikon D3000, Nikon Corporation, Tokyo, Japan). The camera was angled 90 degrees to the skin surface and the pictures were taken from approximately the same distance. A calibrated scale with a code number and letter for each horse and lesion were held to the skin to allow a correct measurement and identification of the wounds. Digital planimetry software (PictZar^® ^CDM, BioVisual Technologies L.L.C. New Jersey, USA) was used for objective wound area calculations (Figure 1). Measurements were taken by the same person throughout the study.

**Figure 2:**
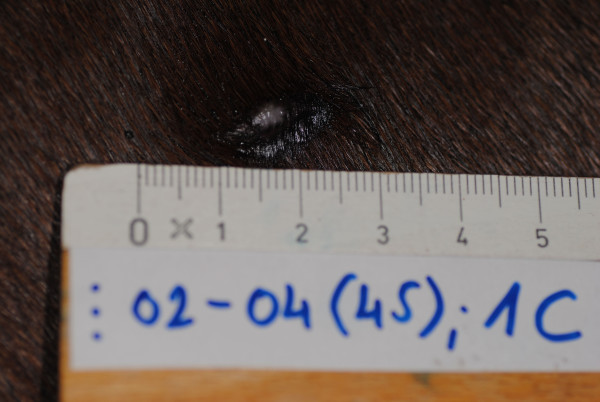
**Wound photographed at the time of complete healing**. The same wound (as on Fig 1a and 1b) is photographed at the time of complete epithelization (healing) at day 45.

After day 28 all unhealed wounds were ocularly examined daily by the same examiners to assess whether complete healing had occurred. The wounds were considered healed when an epithelial layer covered the entire wound surface. The time required for complete healing was recorded and the wound was photographed (Figure 2).

Dependent variables in the statistical analyses were days until healing, area of the wound, subjective scoring of the wound, and cytological scoring. The effect of LHP^®^cream treatment (LHP), petrolatum (P) or untreated (U) on the outcome was analyzed with mixed linear regression methods, where the model included the fixed effects of treatment, examination day, the interaction between treatment and examination day, and the random effect of horse, to account for the repeated observations on horse. However, the model on days until healing only included the effects of treatment and horse. The model fit was evaluated by residual analysis, and all models were considered acceptable with the residuals following a normal distribution and showing no heteroscedasticity. Associations between treatment and results of the bacterial cultures were analyzed with Fisher's exact tests, because the multivariable statistical models did not converge. All wound locations, outcomes and treatment protocols were identified by a code and blinded to the analyst.

## Results

The wounds caused only minimal local discomfort to the horses. Wound areas were mildly swollen and were mildly sensitive on palpation only during the initial days after wounding. All horses had normal rectal temperature during the study period and none of the wounds showed clinical signs of infection such as severe swelling, discharge or pain. Therefore medication with antimicrobials or with nonsteroidal anti-inflammatory drugs was not necessary and all horses could complete the study. Side effects related to wound treatments were not noticed except a slight and transient bleaching of the hairs around LHP^® ^treated wounds.

**Figure 3:**
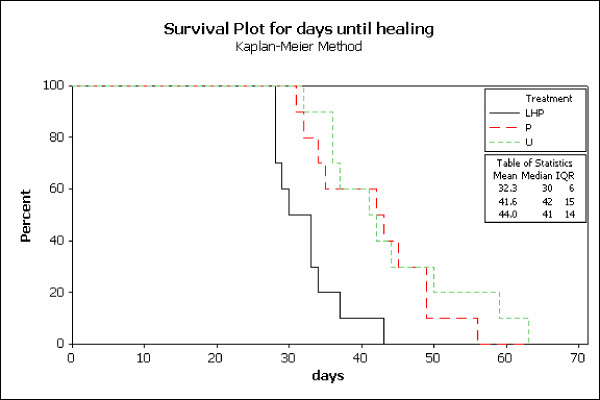
**Kaplan-Meier survival plot of days from start of the study until healing according to treatment**.

**Figure 4:**
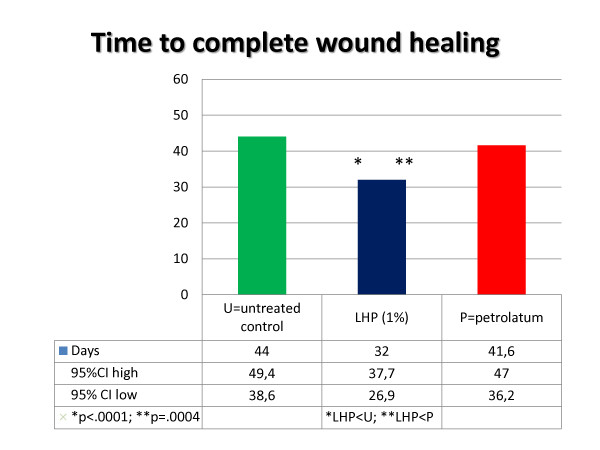
**Histogram of days from start of the study until healing according to treatment**.

The LHP^® ^treated wounds healed significantly faster compared to both petrolatum treated wounds (p = 0.0004) and untreated controls (p < 0.0001). Mean time to complete healing for LHP^® ^treated wounds was 32 days (95%CI = 26.9-37.7). Mean time to complete healing for petrolatum and untreated control wounds was 41.6 days (95%CI = 36.2-47.0) and 44.0 days (95%CI = 38.6-49.4) respectively (Figure 3 and 4). On each individual horse the LHP^®^ treated wound was the first one to heal.

There was no significant difference in area of the three types of wounds at day 0 and day 1 and there was no substantial retraction noted during the first days following wounding. Wound areas decreased significantly faster in untreated wounds compared to LHP^® ^treated and petrolatum treated wounds between day 2 and 6 (p < 0.0001). However, areas of LHP^® ^treated wounds and petrolatum treated wounds were significantly smaller compared to untreated wounds on day 16 (p = 0.0477; p = 0.0021, respectively).

**Figure 5:**
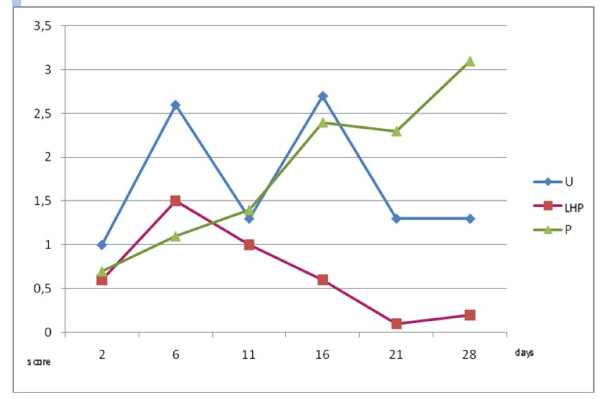
**Cytology scores for bacteria in samples taken within lesions**. Samples are taken on day 2, 6, 11, 16, 21 and 28 (U = untreated controls; LHP = LHP^®^treated wounds; P = petrolatum treated wounds).

**Figure 6:**
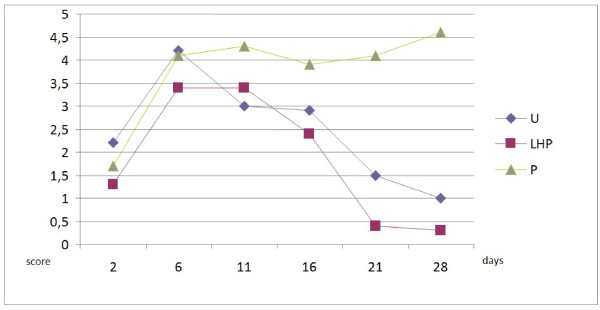
**Cytology scores for neutrophils in samples taken from lesions**. Samples are taken on day 2, 6, 11, 16, 21 and 28 (U = untreated controls; LHP = LHP^®^treated wounds; P = petrolatum treated wounds).

Cytology scores for bacterial counts from samples taken within lesions were significantly lower in the LHP^® ^group compared to untreated controls and petrolatum on day 16 (p < 0.0001 and p = 0.0003 respectively), on day 21 (p = 0.0152 and p < 0.0001) and on day 28 (p = 0.0258 and p < 0.0001) (Figure 5). Untreated controls had significantly higher bacterial counts compared to LHP^® ^treated wounds on day 6 (p = 0.026). There were no significant differences between untreated controls and petrolatum treated wounds except at day 6 (p = 0.0025 and 28 (p = 0.0003). Neutrophil scores were significantly (p < 0.0001) higher for petrolatum treated wounds compared to LHP^® ^treated and untreated controls on day 21 and 28 (Figure 6). Petrolatum treated wounds also had significantly higher neutrophil scores compared to LHP^® ^wounds on day 16 (p = 0.0046) and compared to untreated controls on day 11 (p = 0.0138). Furthermore, the LHP^® ^wounds had lower scores compared to untreated controls (however statistically significant only on day 21, p = 0.0367).

Bacterial culture yielded growth from the vast majority (*n *= 86) of wound samples from the 10 horses, except in 4 samples from 4 horses. Cultures from LHP^®^treated wounds yielded sparse or, in a few samples, no growth in the majority of horses regardless of the day of sampling (day 2, 6 or 16). Petrolatum treated wounds had significantly (p < 0.001) more observations of moderate to profuse growth on day 16 compared to LHP^® ^treated and untreated wounds (Table 1). Growth of a mixed unspecific flora was the most common result.

**Table 1:** Number of positive bacterial cultures from untreated, LHP^®^ treated and petrolatum treated wounds

	Untreated wounds		LHP^®^treated wounds		Petrolatum treated wounds		
		
Sampling Day	1-2	3-4	1-2	3-4	1-2	3-4	p-value (Fisher's exact test)
2	6	4	7	3	10	0	0.15
6	6	4	9	1	8	2	0.43
16	10	0	8	2	1	9	< 0.001

Growth is classified as no growth, sparse, moderate or profuse, on the basis of the number of colonies on the plates, in untreated wounds, wounds treated with LHP^®^cream and petrolatum treated wounds from 10 horses.

1 No growth (0 colonies), 2 sparse growth (1-19 colonies), 3 moderate growth (20 to 100 colonies) and 4 profuse growth (> 100 colonies).

*Staphylococcus aureus *and/or *Streptococcus zooepidemicus *were isolated from petrolatum treated and untreated wounds but not from LHP^® ^treated wounds. Petrolatum treated wounds had significantly (p < 0.001) more observations of growth for *S. aureus *and/or *S. zooepidemicus *on day 16 compared to LHP^® ^treated and untreated wounds (Table 2).

**Table 2:** Positive cultures for *Staphylococcus aureus *and/or *Streptococcus zooepidemicus *in untreated, LHP^®^treated and petrolatum treated wounds

No of horse positive for *S. aureus *and/or *S. zooepidemicus* (No of investigated horses)				
**Sampling day**	**Untreated wounds**	**LHP^®^treated wounds**	**Petrolatum treated wounds**	**p-value (Fisher's exact test)**

2	0 (10)	0 (10)	0 (10)	-
6	2 (10)	0 (10)	3 (10)	0.32
16	0 (10)	0 (10)	7 (10)	< 0.001

** LHP^®^cream base (w/w %): Glycerol monolaurate (7.0); Glyceryl monomyristate (21.0); Polyoxyethylene (100)stearate (1.0); Propylenglycol (2.0); Citronsyra (0.9); Natriumhydroxid to ph 4.5-4.8 (0.3); Sulfuric acid 1 M (0.04); Sodium oxalate (0.14); Salicylic acid (0.1); Sodium edetate (0.05); Sodium pyrofosfate (0.025); Sodium stannate (0.04); water (100.0)

Wounds treated with LHP^® ^received significantly lower summarized subjective scores (evaluating swelling, sensitivity to the touch, discharge, granulation and epithelization) compared to petrolatum treated wounds on day 21 and 28 (p < 0.0001). Furthermore LHP^® ^treated wounds received significantly lower scores compared to untreated wounds on day 6, 16, 21, and 28 (P = 0.0007-0.0064). Petrolatum treated wounds received significantly lower scores compared to untreated ones on day 11 and 16 (p = 0.0121; p = 0.0121), but on day 28 petrolatum treated wounds had significantly higher scores (p = 0.0362).

Untreated wounds produced less exudate compared to both LHP^® ^and petrolatum treated wounds and a hard crust covered the wound surface until the wound was completely healed. Moderate amounts of exudates was produced in LHP^® ^treated wounds and crust partially covered the areas, whereas petrolatum treated wounds accumulated the most exudate and had minimal or no crusting. Petrolatum treated wounds showed excessive granulation, where the granulation tissue protruded (grossly in the centre of the wound) over the level of the surrounding skin but did not overlap wound edges. Exuberant granulation tissue formation was not observed in any LHP^® ^or untreated wounds. Hair appeared to grow slightly faster along petrolatum treated wound edges. Wounds became slightly oval after surgery and grossly maintained this shape until the end of healing regardless of treatment. Most wounds developed a circular scar but some wounds became elongated in the final period of healing and developed linear scarring.

## Discussion

Hydrogen peroxide cream (LHP^®^) treated wounds healed significantly faster compared to petrolatum treated wounds and untreated controls and on all horses the LHP^® ^treated wound was the first one to heal.

Wound healing could be influenced by a number of factors. Fibroblasts have an essential role in the proliferation phase of wound healing and also in wound contraction [22,23]. Although 3% hydrogen peroxide in water solution was found cytotoxic when directly applied to cultured human fibroblasts [24], it has been described to have positive effects on fibroblast proliferation and activity in low concentrations [25-28]. Hydrogen peroxide has also been associated with vascular endothelial growth factor release [29] and increased wound contraction via angiotensin II [30]. It could be speculated that in the present study a release of hydrogen peroxide from the LHP^® ^cream resulted in a continuous low level hydrogen peroxide concentration in the wound areas which could have a positive effect on the intensity of granulation and wound contraction without being toxic on proliferating cells.

Tissue vascularisation is a factor of critical importance for wound healing. In treatment of ischemic ulcers in guinea pigs, hydrogen peroxide cream (1.5%; 2% and 3.5%) was shown to increase blood flow in wound areas and surrounding skin. Furthermore, vascularisation dose dependently increased when higher concentration of hydrogen peroxide was used [18]. It is possible that an increased blood flow in the wounds induced by the hydrogen peroxide in the LHP^® ^cream could have contributed to a faster healing, although we did not investigate circulatory effects.

The shorter healing time of LHP^® ^treated wounds could also be due to a beneficial effect of components of the cream base itself. Of the components propylene glycol has been investigated on second intention wound healing in horses. However, in that study propylene glycol did not have any effect on the wound healing process [31].

Moist environment, low pH and high oxygen tension are expected to facilitate wound healing [32]. These factors could all have some positive impact on healing time when using LHP^® ^cream (pH 4.5) on the wounds. However, these effects were not investigated in the present study.

It has been suggested that hydrogen peroxide in water solution could impair normal epithelisation causing bullae and ulceration on newly epithelised wounds [33]. In our study LHP^® ^cream treatment did not generate any noticeable disturbance in normal wound epithelisation and the horses did not experience any irritation or other negative effects on surrounding skin.

Bacterial infection has been associated with delayed wound healing [1]. Bacterial colonisation of open wounds might contribute to increased risk of infection, thus cytology samples and bacterial cultures were taken from the wound areas during the study. Hydrogen peroxide has been reported to be effective against both Gram negative and Gram positive bacteria [34] and in a cream formula it was shown to have excellent antibacterial properties in the treatment of the human infectious skin disease impetigo contagiosa [15]. It also occurs naturally in the body and is released by phagocytising neutrophils during early inflammatory processes to kill bacteria [35,36]. We found significantly lower bacterial counts on cytology in LHP^® ^treated wounds compared to petrolatum and untreated controls from day 16. Also petrolatum treated wounds yielded significantly higher number of cultures with moderate to profuse growth at day 16 compared to LHP^® ^treated and untreated controls. Furthermore untreated controls had significantly higher bacterial counts on cytology compared to LHP^® ^treated wounds on day 6 (p = 0.026) and although there was no statistically significant difference in the intensity of bacterial growth in cultures between LHP^® ^wounds and untreated controls, the potential pathogens, *S. aureus *and *S. zooepidemicus*, were only found in untreated or petrolatum treated wounds. These results suggest that LHP^® ^cream decreased bacterial growth in wounds, which is possibly explained by the antibacterial effect of hydrogen peroxide component. However, components of the cream base itself could also have contributed to the antibacterial effect associated with the treatment of the LHP^® ^cream. For example propylene glycol and monoglycerides have been reported to have antibacterial properties [37,38].

An adequate inflammation is important for optimal wound healing, but a prolonged inflammatory phase has a delaying effect [39]. Reason for prolonged inflammation may include the presence of bacteria. In this study cytology scores for neutrophils were significantly lower for wounds treated with LHP^® ^cream compared to those receiving petrolatum. The lower number of neutrophils present in LHP^® ^treated wounds from day 16 can indicate a shorter inflammatory phase.

In the present study no obvious wound retraction was noticed during the initial period after wounding, which is similar to previously described wounds on the body in ponies [40]. However in that study identical sized (20 mm diameter) untreated, circular, full thickness skin wounds healed faster (25 ± 3.5 days) than the untreated wounds in the present study (44.0 days; 95%CI = 38.6-49.4). This could support earlier observations that wounds in ponies heal faster compared to wounds in horses [41,42].

We created wounds on the sides of the neck because wounds on the body in horses expected to heal significantly faster and with less exuberant granulation tissue formation compared to wounds on the distal limb [32,40,41]. However we experienced that petrolatum treated wounds developed excessive granulation tissue. One possible explanation could be the occlusive properties of petrolatum, creating lower oxygen tension which could result in excessive granulation tissue growth [43]. The degree of excessive granulation did however not require any additional treatment. Granulation tissue did not impair accurate wound area measurements and did not seem to impair wound healing. In fact petrolatum treated wounds seemed to heal somewhat faster than untreated controls.

Petrolatum did not seem to impair wound healing in this study, but it induced excessive granulation and petrolatum treated wounds accumulated significantly more bacteria. Also, petrolatum treated wounds had significantly more observations of growth for *S. aureus *and/or *S. zooepidemicus *compared to LHP^® ^treated and untreated wounds. Therefore petrolatum for topical treatment of open wounds in horses may not be an advisable alternative, especially on the distal limb where the tendency for excessive granulation and the risk for bacterial contamination is high [40].

Bacterial colonization was evaluated by taking bacterial cultures and also by swabs for cytology in order to count bacteria. Samples were taken by rolling sterile swabs on the surface of wounds following a standardized protocol, identical in each wound. A surface sample is not representative for the depth of the wound but it gives sufficient information on surface colonization with minimal interruption of wound healing. Biopsies could have given more information on the depth but only in localised areas and would also have interrupted wound healing. Sampling and wound handling (treatment and cleansing) were designed to be identical in all wounds and most importantly, minimally interfere with wound healing. Swabs were rolled in the same pattern in all wounds, wounds were cleaned by the same amount of moistened swabs and crusts were not removed.

The paired wounds were randomly chosen to be treated in order to evaluate the effect of the two different treatments on wounds at identical anatomical locations. Also the treated wounds were on the opposite side of the neck to prevent interaction between the different treatments. The third, untreated control wounds were placed at the neck where the circulatory support of the skin, and subcutaneous tissue characteristics were judged to be identical or at least very similar to the areas of the treated wounds. The distance between treated and untreated wounds was estimated to prevent interaction between the creams and the untreated wound areas.

Digital techniques such as photoplanimetry have been used to monitor wound healing by recording wound area changes in the daily wound care in human hospitals and it has been used in several wound healing studies as well [44-47]. In this study the even and perpendicular surface of the neck and the relatively small and uncomplicated wounds allowed to take realistic photos and facilitated accurate wound area measurements.

In this experimental study a beneficial effect of LHP^® ^(1% hydrogen peroxide) cream on wound healing was shown. However, we do not know whether this effect could be associated with the hydrogen peroxide content of the cream or the cream base itself. To investigate this, further experimental studies are needed to compare the effect of LHP^® ^cream to the cream base on wound healing.

## Conclusion

Treatment with LHP^® ^cream was associated with earlier complete wound healing and with reduced bacterial colonization compared to untreated or petrolatum treated wounds. No adverse effects of LHP^® ^cream were noticed. The use of LHP^® ^cream appears to be safe and effective for topical wound treatment and/or protection. In this study petrolatum was less beneficial for wound healing and was associated with increased bacterial colonization.

## Competing interests

The authors declare that they have no competing interests.

## Authors' contributions

TT: Main initiator, designer and performer of the project and author of the manuscript; HB: Contributor to design and revising the manuscript, acquisition of funding; VB and EB: Bacterial cultures and analyses; UE: Statistical analyses; TK: Assistant performer and author and KB: Main coordinator, contributor to conception and design, cytological analysis, revision of the manuscript. All authors read and approved the final manuscript.
